# Modulation of experimental atopic dermatitis by topical application of Gami-Cheongyeul-Sodok-Eum

**DOI:** 10.1186/1472-6882-13-312

**Published:** 2013-11-11

**Authors:** Ji Sun Hwang, Jung-Eun Kim, Young-Beob Yu, Sin-Hyeog Im

**Affiliations:** 1 School of Life Sciences and Immune Synapse Research Center, Gwangju Institute of Science and Technology (GIST), Gwangju 500-712, Republic of Korea; 2 Department of Herbal Pharmaceutical Development, Nambu University, 23, Cheomdan-jungangro, Gwangsan-gu, Gwangju 506-706, Republic of Korea

**Keywords:** Atopic dermatitis, Herbal medicine, GCSE (Gami-Cheongyeul-Sodok-Eum), Th1 & Th2 cells, B cells, IgE and Cytokines

## Abstract

**Background:**

Gami-Cheongyeul-Sodok-Eum (GCSE), an herbal formula of traditional Korean medicine, comprises nine herb components. GCSE has various biological activities such as anti-inflammatory, anti-bacterial and anti-viral activities. However, it is still unclear whether GCSE has any immunomodulatory effect on atopic dermatitis (AD).

**Methods:**

GCSE was treated to primary B cells and CD4+ T cells isolated from atopic mice to compare its inhibitory effects on IgE secretion and cytokine expression. Experimental AD was established by alternative treatment of 2, 4-dinitrochlorobenzene (DNCB) and house dust mite extract to the ears of BALB/c mice. GCSE was topically applied to ears of atopic mice every day for 3 weeks. AD progression was analyzed by measuring ear thickness, serum IgE level, histological examination of ear tissue by H&E staining and cytokine profile of CD4+ T cells and CD19+ B cells by real time PCR and ELISA.

**Results:**

Treatment of GCSE significantly reduced IgE production and expression of AD associated pathogenic cytokines such as IL-4, IL-5, IL-10, IL-13, IL-17, TNF-α, and IFN-γ by lymphocytes isolated from AD-induced mice. Topical application of GCSE on the ears of AD-induced mice significantly reduced ear thickness, clinical score and lymphocytes infiltration to ears as compared to control group. GCSE treatment also reduced serum IgE level and the levels of major pathogenic cytokines such as IL-4, IL-5, IL-10, IL-13 and IL-17. In addition, GCSE treatment significantly increased Foxp3 expression level.

**Conclusions:**

The protective effect of GCSE in experimental AD is mediated by inhibition of IgE production, by reduction in the levels of pathogenic cytokines and by induction of Foxp3, all of which are suggesting the beneficial effect of GCSE on modulating atopic dermatitis.

## Background

Atopic dermatitis (AD) is a chronic relapsing skin disease that is manifested by Th2 dominant hyperimmune disorder, the incidence of which has rapidly increased especially in the industrialized countries [1,2]. AD is caused by complex pathogenic factors including genetic susceptibility, host’s environment, skin barrier dysfunction, bacterial infection and immunological factors [3,4]. The major symptoms of AD are severe scratching, pruritus, dryness and inflammation [1,5], which are mediated by Th1 and Th2 immune responses. Th2 cells produce IL-4, IL-5, and IL-13 and play major roles in acute atopic dermatitis [6-8]. Enhanced circulating IgE levels in AD patients are mainly caused by increased production of IL-4 and IL-13 [9,10]. In the later stage of AD where infection mediated inflammation occurs, Th1-type cytokines such as IFN-γ, and IL-12 mediate the chronic symptoms of atopic dermatitis [11-13]. Association of both Th1 and Th2 immune response in AD pathogenesis makes it hard to successfully treat AD patients. Although topical steroid therapy using corticosteroid or calcineurin inhibitor is widely used for AD treatment, it has diverse side effects. Recently, natural herbs or oriental medicines are employed as new treatments for AD modulation based on their potent disease-modifying efficacy with less side effects [14,15].

Gami-Cheongyeul-Sodok-Eum (GCSE) is a modified formula of Cheongyeul-Sodok-Eum that has anti-inflammatory and anti-allergic effects [16,17]. GCSE is a mixture of nine kinds of oriental medicine extracts comprising of *Angelicae Gigantis Radix, Astragali Radix, Atractylodis Rhizoma Alba, Coptidis Rhizoma, Forsythiae Fructus, Glycyrrhizae Radix, Lonicerae Flos, Portulacae Herba* and *Scutellariae Radix. Angelicae Gigantis Radix* exhibits the anti-inflammatory function through the inhibition of NFκB dependent pro-inflammatory cytokine expression [18]. Decursin, a major component of *Angelicae Gigantis Radix*, modulates inflammation by inhibiting NFκB-Cox-2-PGE2 mediated pathways [19]. Arctigenin, a bioactive agent of *Forsythiae Fructus,* has anti-inflammatory action through the inhibition of leukocytes exudation and recruitment into the inflamed tissues. Extract of *Astragali Radix* has anti-inflammatory effect by inhibiting the activation of p38 and Erk1/2 and NFκB-mediated transcription [20]. However, no investigation has been performed to evaluate the AD modifying activity of GCSE especially upon topical application.

In the present study, we examined the therapeutic effects of GCSE on experimental AD and elucidated its action mechanism. *In vitro* treatment of GCSE to the lymphocytes isolated from AD-induced mice suppressed IgE production and significantly reduced the levels of pathogenic cytokines. In addition, topical application of GCSE to the mice with ongoing atopic dermatitis significantly suppressed AD progression by down-regulating the levels of pathogenic cytokines and serum IgE levels.

## Methods

### Standardization of Gami-Cheongyeul-Sodok-Eum (GCSE)

The preparation of Gami-Cheongyeul-Sodok-Eum (GCSE) was performed in compliance with the test guidelines of the Korea Food and Drug Administration (KFDA). The GCSE was prepared as described in Table 1. The 9 herbs used in the GCSE were purchased from Gwang Myung Dang Pharmaceutical Company (Ulsan, Korea), identified by Prof. Bu, Department of Oriental Medicine, Kyunghee University, and were authenticated by the Jeonnam Traditional Korean Medical Institute (Jangheung, Korea) based on the Korean pharmacopoeia guidelines. All herbal voucher specimens (2010-GCSE-01 ~ GCSE-09) in GCSE were deposited at the Department of Herbal Pharmaceutical Development (Nambu University, Gwangju, Korea). They were ground into powder (135.0 g, 80 mesh), and were extracted with 1,350 mL of 70% aqueous ethanol at 80°C. The crude extract was concentrated and lyophilized in vacuo. The weight of the final GCSE extract was approximately 29.6 g (21.9% of the starting raw herbs). Each herb was tested for heavy metal (Hg, As, Cd) contamination, residual insecticides, and microbial limit including LPS contamination. All the materials under study are endotoxin-free. Standardization of each herb extract was performed by high performance liquid chromatography (HPLC) analysis. The content of marker substances in herb extract was compared with commercially available indicator chemicals; glycyrrhizin, liquiritigenin, baicalin, baicalein, wogonin and berberine from Wako Pure Chemical Industries, Ltd. (Osaka, Japan); decursin and nodakenin from Korea Food and Drug Administration (KFDA, Seoul, Korea). Other chemicals were of analytical grade. A Shimadzu LC 20 AD (Shimadzu, Japan) consisting of quaternary solvent blending, Sil 20A autosampler, column heater, and SHIMADZU SPD-M20A diode array detector was used to perform HPLC analysis. The dried GCSE was kept at 4°C before use.

**Table 1:** Botanical names and origin of the formula, Gami-Cheongyeul-Sodok-Eum

**Systematic name (Pharmaceutical name, sample number)**	**Amount (g)**	**Contents of Marker substances**
*Angelica gigas* (Angelica Gigantis Radix, C1)	1.5	0.44% of nodakenin
		4.22% of decursin
		3.0% of decursinol angelate
*Astragalus membranaceus* (Astragali Radix, C2)	1.5	
*Atractylodes japonica* (Atractylodis Rhizoma Alba, C3)	1.5	
*Coptis japonica* (Coptidis Rhizoma, C4)	1.5	6.80% of berberine
*Forsythia virdissima (*Forsythiae Fructus, C5)	1.5	
*Glycyrrhiza uralensis* (Glycyrrhizae Radix, C6)	1.5	3.04% of glycyrrhizin 0.87% of liquiritigenin
*Lonicera japonica (*Lonicerae Flos, C7)	1.5	
*Portulaca oleracea (*Portulacae Herba, C8)	1.5	
*Scutellaria baicalensis* (Scutellariae Radix, C9)	1.5	15.80% of baicalin
		0.10% of baicalein
		0.04% of wogonin
Total amount	13.5	

### Cytotoxicity examination by WST-1 assay

Cytotoxicity of GCSE was conducted using EZ-Cytox cell viability assay kit (Daeil Lab Service Co, Korea). The manufacturer’s protocol was followed. In summary, 5 × 10^3^ cells/well isolated from spleen were dispensed in a 96-well plate and incubated for 24 hrs. Various concentrations of GCSE, dissolved in 70% ethanol, were treated to the cells and were incubated for 72 hrs. Then cells were incubated with 10 μl of the same reagent for 4 hrs. Using the microplate reader, the absorbance of the soup was measured at 450 nm. Data were presented by relative growth inhibition to GCSE non-treated cells.

### Animals and Induction of atopic dermatitis

Female BALB/c mice (6–8 weeks) were purchased from SLC Inc. (Hamamatsu, Japan) and female Foxp3-GFP knock in mice (6–8 weeks) were purchased from The Jackson Laboratory (CA, USA). Mice were housed in specific pathogen-free barrier facility. All experimental procedures were performed in accordance with the Guidelines of National Animal Welfare Law of Korea for the care and use of laboratory animals and were approved by Animal Care and Ethics Committees of the Gwangju Institute of Science and Technology (GIST) (permit number: GIST-2011-3). Induction of experimental atopic dermatitis was performed as previously described [21]. The surfaces of both ear lobes of mice were stripped with surgical tape (Nichiban, Tokyo, Japan). After stripping, 20 μl of 2% 2, 4-dinitrochlorobenzene (DNCB) (Sigma Aldrich, St Louis, MO, USA) dissolved in acetone/olive oil solution (acetone: olive oil = 1:3) was painted on each ear. After 3 days, 150 μg of mite extract (*Dermatophagoides farinae*, GREER source materials, Lenoir, NC, USA) dissolved in PBS containing 0.5% tween 20, was re-painted on ears of mouse. Challenge of DNCB and mite extract was alternately repeated once a week for 6 weeks. After 3 weeks of AD induction, mice were divided into three groups based on similarity of AD severity clinical scores. Then, mice in each group were painted daily with 70% ethanol (Cont), GCSE-2 mg, or GCSE-10 mg on both ears for additional 3 weeks while continuously inducing atopic dermatitis.

### Measurement of ear swelling

Ear thickness was measured 24 hrs after application of DNCB or mite extract with a dial thickness gauge (Kori Seiki MFG, Co., LTD., Japan). A representative mouse of each group was photographed to show the clinical symptoms.

### Histological examination

Excised ears of each group were fixed in 4% paraformaldehyde for 16 hrs and were embedded in paraffin. Then, 6 μm sections were stained with hematoxylin (Sigma Aldrich, St. Louis, MO, USA) and eosin (Sigma Aldrich, St. Louis, MO, USA) (H&E). Infiltrating lymphocytes, thickening of the epidermis, and fibrosis in the dermis were observed by microscope (50X, 100X, 200X).

### ELISA

Total IgE levels in the serum were measured using sandwich ELISA kit (BD Biosciences) following the manufacturer’s protocol. For the detection of IgE production from B cells, CD19+ B cells isolated from AD-induced mice were treated with diverse concentrations of GCSE, and IgE levels were measured by ELISA (BD Biosciences, San Diego, CA, USA). For the detection of cytokine concentration (IL-4, IL-10, IL-17, and IFN-γ) in the culture supernatant, ELISA was performed by using ELISA kits (e-bioscience, San Diego, CA, USA).

### Isolation of primary CD4+ T cells and CD19+ B cells

Draining lymph nodes (superficial cervical, axillary, and brachial lymph nodes) from mice were ground using cell strainer (BD Biosciences, San Diego, CA, USA). CD19+ B cells or CD4+ T cells were isolated using magnetic beads (Miltenyi Biotech, Germany) according to the manufacturer’s protocol [21,22].

### RNA isolation, quantitative RT-PCR (qRT-PCR)

For the cytokine analysis, 3 x 10^6^ cells of CD4+ T cells or CD19+ B cells from each group were stimulated with PMA (50 ng/ml)/ionomycin (1 μM) and LPS/IL-4 (10 μg/ml) for 4 hrs, respectively. Total RNA was extracted from stimulated cells with TRIzol reagent (Molecular Research Center, Cincinnati, OH, USA) according to manufacturer’s protocol. For reverse transcription, 1 μg of total RNA was used. To generate cDNA, oligo (dT) primer (Promega, Madison, WI, USA) and Improm-II reverse transcriptase (Promega, Madison, WI, USA) with a total volume of 20 μl were used. The mRNA level was determined using 1 μl of cDNA by real time PCR with SYBR using a protocol provided by the manufacturer (MJ research chromo4). Mouse HPRT primer was used for qRT-PCR to normalize the amount of cDNA used for each condition. PCR was performed with the following primers: HPRT (Forward - 5’ TTA TGG ACA GGA CTG AAA GAC 3’ , Reverse - 5’ GCT TTA ATG TAA TCC AGC AGG T 3’); IL-4 (Forward - 5’ ACA GGA GAA GGG ACG CCA T 3’ , Reverse - 5’ GAA GCC GTA CAG ACG AGC TCA 3’); IL-5 (Forward - 5’ AGC ACA GTG GTG AAA GAG AC 3’ , Reverse - 5’ TCC AAT GCA TAG CTG GTG ATT T 3’); IL-10 (Forward - 5’ ATA ACT GCA CCC ACT CCC A 3’ , Reverse - 5’ TCA TTT CCG ATA AGG CTT GG 3’); IL-13 (Forward - 5’ GCA ACA TCA CAC AGG ACC AGA 3’ , Reverse - 5’ GTC AGG GAA TCC AGG GCT AC 3’); IL-17A (Forward - 5’ TTC ATC TGT GTC TCT GAT GCT 3’ , Reverse - 5’ TTG ACC TTC ACA TTC TGG AG 3’); IFN-γ (Forward - 5’ GAG CCA GAT TAT CTC TTT CTA CC 3’ , Reverse - 5’ GTT GTT GAC CTC AAA CTT GG 3’); TNF-α (Forward - 5’ CCC TCA CAC TCA GAT CAT CTT CT 3’ , Reverse - 5’ GCT ACG ACG TGG GCT ACA G 3’); Foxp3 (Forward - 5’ TTC CTT CCC AGA GTT CTT CC 3’ , Reverse - 5’ CTC AAA TTC ATC TAC GGT CCA 3’).

### Reagents and cell culture

The isolated primary cells were cultured in RPMI 1640 medium (Welgene, Daegu, Korea) supplemented with 10% fetal bovine serum (Hyclone, USA), 3 mM L-glutamine (Sigma Aldrich, St Louis, MO, USA), 100 U/ml penicillin (Sigma Aldrich, St. Louis, MO, USA), 100 U/ml streptomycin (Sigma Aldrich, St. Louis, MO, USA), non-essential amino acids (Welgene, Daegu, Korea), sodium pyruvate (Welgene, Daegu, Korea), HEPES (Welgene, Daegu, Korea) and 0.05 mM 2-beta-mercaptoethanol (Sigma Aldrich, St Louis, MO, USA). For the cytokine analysis in AD experiments, cells were stimulated with PMA (50 ng/ml) and ionomycin (1 μM) or LPS (10 μg/ml) for 4 hrs. In order to perform the ELISA, cells were stimulated with LPS (10 μg/ml)/IL-4 (5 ng/ml) for 72 hrs.

### *In vitro* iTreg generation

CD4+ T cells isolated from the spleen and lymph node of 8 weeks old Foxp3-GFP knock-in mice were stimulated in a medium supplemented with anti-CD3 (1 μg/ml) / CD28 Ab (3 μg/ml), anti-IL-4 Ab (10 μg/ml), anti-IFN-γ Ab (10 μg/ml), and TGF-β (5 ng/ml) at day 1 and additional 50 U/ml of rhIL-2 at day 3. Then, iTreg cells were stimulated with various concentrations of GCSE in the presence of PMA (50 ng/ml)/ ionomycin (1 μM) for 12 hrs. Relative mRNA expression levels of Foxp3 of GCSE treated samples were compared with control sample by qRT-PCR and protein level of Foxp3 was measured by flow cytometry.

### Statistical analysis

A Student's t-test was used to calculate the statistical significance of the experimental data. The level of significance was set at *P < 0.05, **P < 0.01 and ***P < 0.001. Significance was only indicated when appropriate.

## Results

### Analysis of marker substances in herbs by HPLC

**Figure 1:**
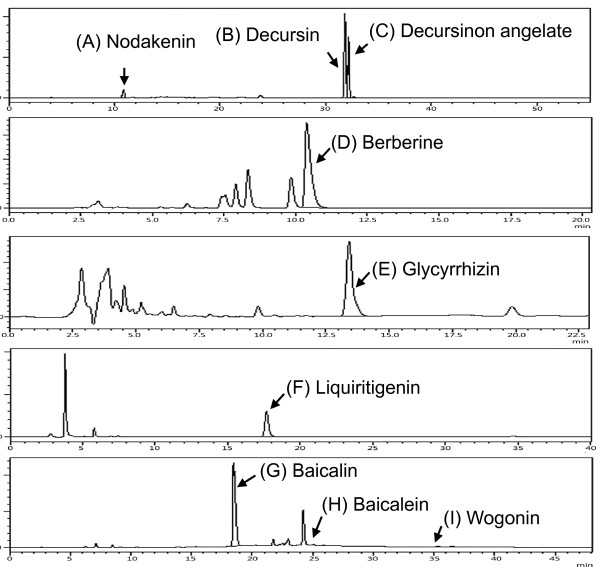
**HPLC chromatograms of marker substances in individual herbs of Gami-Cheongyeul-Sodok-Eum (GCSE).** HPLC analysis was performed on YMC-Pack Pro C18 column (4.6 × 250 mm, 5 μm). The optimum mobile phase and wavelength for detection were acetonitrile and water for **(A)** nodakenin, **(B)** decursin and **(C)** decursinol angelate (gradient, 20% - 80% ACN, 330 nm); **(D)** acetonitrile and water (3.4 g of monobasic potassium phosphate and 1.7 g of sodium lauryl sulfate/1000 mL) for berberine (1:1, 345 nm); aqueous acetic acid and acetonitrile for **(E)** glycyrrhizin (3:2, 254 nm) and **(F)** liquiritigenin (7.5:2.5, 276 nm); aqueous acetic acid and acetonitrile : methanol (7:3) for **(G)** baicalin, **(H)** baicalein, and **(I)** wogonin (gradient, 25% - 52% ACN-MeOH, 277 nm). Data are representative of three independent experiments.

To ensure the quality and purity of each preparation of GCSE, HPLC analysis was performed by measuring the content of known active compounds of the nine marker substances of four herbs of GCSE by following the Korean Pharmacopoeia Guidelines (9^th^ edition). Decursin, decursinol angelate and nodakenin in *Angelicae Gigantis Radix* were quantified by HPLC-DAD using a C18 column (YMC-Pack Pro, 4.6 × 250 mm, 5 μm) and gradient elution with water and acetonitrile. The amount of decursin, decursinol angelate, and nodakenin in *Angelicae Gigantis Radix* were calculated as 4.22, 3.00 and 0.44%, respectively. The contents of marker substances in *Coptidis Rhizoma* (6.80% berberine)*, Glycyrrhizae Radix* (3.04% glycyrrhizin, 0.87% liquiritigenin), and *Scutellariae Radix* (15.80% baicalin, 0.10% baicalein, 0.04% wogonin) were calculated (Table 1 and Figure 1). These results indicate that the content of these nine compounds in the GCSE showed the upper value of the contents criterion in Korean Pharmacopoeia Guidelines (9^th^ edition).

### Effect of GCSE treatment on T cells and B cells isolated from AD-induced mice

**Figure 2:**
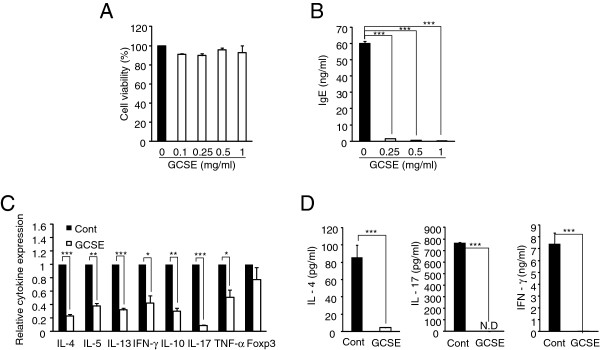
**Effect of GCSE treatment on T cells and B cells isolated from AD-induced mice. (A)** Mouse splenocytes were incubated with various concentrations of GCSE for 72 hrs. Cell viability was estimated with WST-1 assay. **(B)** Draining lymph node CD19+ B cells isolated from AD-induced mice were stimulated with LPS (10 μg/ml)/IL-4 (5 ng/ml) in the presence of various concentrations of GCSE dissolved in 70% alcohol for 72 hrs. Mouse IgE levels in the supernatant of B cell culture were measured by ELISA. Same volume of 70% alcohol was treated as control. **(C)** Draining lymph node CD4+ T cells from AD-induced mice were stimulated with PMA (50 ng/ml)/ionomycin (1 μM) in the presence of GCSE (0.25 mg/ml) for 4 hrs. Relative expression of cytokines of GCSE treated samples was compared with control samples by qRT-PCR. Expression level of HPRT was used as an internal control. **(D)** CD4+ T cells from AD-induced mice were stimulated with PMA /ionomycin for 72 hrs in the presence of GCSE (0.25 mg/ml), then protein level of each cytokine was analyzed by ELISA. Error bars indicate SD. One (*), two (**) and three (***) indicate p < 0.05, p < 0.01, and p < 0.001 respectively. Data are representative of three independent experiments.

Determination of optimal concentration of GCSE that does not show cytotoxicity was performed using WST-1 assay. Treatment of GCSE to splenocytes for 72 hrs with up to 1 mg/ml did not induce cell death (Figure 2A). Based on this result, we used 0.25 mg/ml of GCSE or each component of GCSE for all the *in vitro* experiments. In *in vivo* AD condition, we examined the effect of the GCSE treatment on the production of IgE by CD19+ B cells isolated from AD-induced mice. Upon LPS/IL-4 stimulation, GCSE treatment significantly reduced IgE production by B cells in a dose dependent manner (Figure 2B). Then, we also evaluated the effect of the GCSE treatment on the expression level of key cytokines associated with the development of atopic dermatitis. CD4+ T cells isolated from draining lymph nodes of AD-induced mice were stimulated by PMA/ionomycin for 4 hrs in the presence or absence of GCSE (0.25 mg/ml) and the expression levels of cytokine genes were analyzed by qRT-PCR. Treatment of GCSE significantly decreased the expression levels of AD-associated pathogenic cytokines (IL-4, IL-5, IL-13, INF-γ, IL-10, IL-17 and TNF-α) (Figure 2C). In accordance with mRNA result, treatment of GCSE also significantly reduced the protein level of IL-4, IL-17 and IFN-γ in the T cell culture supernatant (Figure 2D). Collectively, these data indicate that treatment of GCSE could inhibit the production of AD-associated pathogenic molecules produced by CD4+ T cells and IgE levels by CD19+ B cells.

### Suppression of AD progression by topical application of GCSE

**Figure 3:**
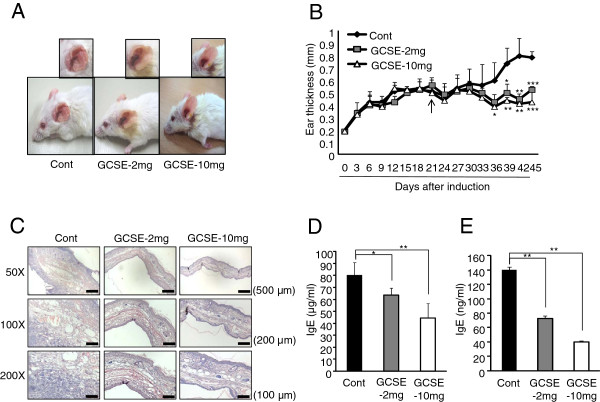
**Inhibition of AD progression by topical application of GCSE. (A)** Representative ear pictures of control group or GCSE treated groups were shown. **(B)** AD progression was assessed by measuring ear thickness, 24 hrs after each DNCB or mite extract treatment. **(C)** Ears excised from each group were fixed with 4% paraformaldehyde for 24 hrs and embedded in paraffin. Paraffin embedded ears were sectioned into 6 μm and stained with hematoxylin and eosin (H&E). Infiltration by lymphocytes and thickness of epidermis were observed under the microscope (50X, 100X and 200X). **(D)** Total serum IgE levels were measured by ELISA, left. CD19+ B cells isolated from draining lymph node of each group were stimulated with LPS (10 μg/ml)/IL-4 (5 ng/ml) for 72 hrs then IgE levels in the culture supernatant were measured by ELISA, right. Error bars indicate SD. One (*), two (**) and three (***) indicate p < 0.05, p < 0.01 and p < 0.001 respectively. Data are representative of three independent experiments.

Down-regulation of IgE production and pathogenic cytokines by *in vitro* GCSE treatment led us to test whether topical application of GCSE could also suppress the AD progression. Experimental AD was induced on both ears of BALB/c mice by alternating challenge with DNCB and house dust mite extract [21,22]. AD symptoms including erythema, horny substance, dryness, and swelling were evidently seen in control group (Figure 3A). However, treatment of GCSE (2 mg or 10 mg) significantly reduced AD symptoms (Figure 3A). In agreement with phenotypic observation, GCSE treatment significantly decreased ear thickness (Figure 3B) as compared with control treatment. Histological analysis further confirmed the therapeutic effect of GCSE. In correlation with reduced thickness of epidermis, the numbers of infiltrating lymphocytes in ear regions were significantly reduced by GCSE treatment as compared with the control group (Figure 3C). Since increased serum IgE level is closely correlated with clinical symptoms of AD, we tested whether improved AD symptom by GCSE treatment is also related with changes in serum IgE levels. In comparison with the control group, topical application of GCSE significantly decreased IgE levels in the serum (Cont; 80.4 μg/ml, GCSE-2 mg; 63.4 μg/ml, GCSE-10 mg; 44.5 μg/ml) (Figure 3D). To investigate whether GCSE treatment could suppress IgE production by primary B cells, CD19+ B cells isolated from the draining lymph nodes of each treatment group were stimulated with LPS/IL-4 for 72 hrs, then secreted IgE level was analyzed using ELISA. As shown in Figure 3E, GCSE treatment significantly reduced IgE expression (GCSE-2 mg; 72.7 ng/ml, GCSE-10 mg; 40.2 ng/ml) as compared with the control group (140.5 ng/ml). These results indicate that topical treatment of GCSE decreases IgE production in the activated B cells.

### GCSE treatment suppresses the levels of pathogenic cytokines

**Figure 4:**
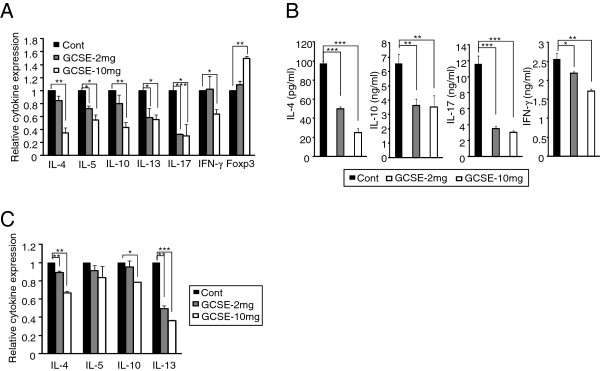
**Down-regulation of pathogenic cytokines by GCSE treatment. (A)** Draining lymph node CD4+ T cells from each treatment group were stimulated with PMA (50 ng/ml)/ionomycin (1 μM) for 4 hrs. Relative cytokine levels of GCSE treated samples were compared with control samples by qRT-PCR. The expression level of HPRT was used as an internal control. **(B)** Draining lymph node CD4+ T cells from each group were stimulated with PMA/ionomycin for 72 hrs, then, IL-4, IL-10, IL-17, and IFN-γ levels were measured by ELISA. **(C)** Draining lymph node CD19+ B cells from each treatment group were stimulated for 4 hrs. Relative cytokine levels of GCSE treated samples were compared with control samples by qRT-PCR. The expression level of HPRT was used as an internal control. Error bars indicate SD. One (*), two (**), and three (***) indicate p < 0.05, p < 0.01 and p < 0.001 respectively. Data are representative of three independent experiments.

Dysregulated cytokine expression in CD4+ T cells mediates the AD pathogenesis [12,23]. We tested whether protective effect of GCSE treatment is also related with changes in cytokine profiles. CD4+ T cells isolated from draining lymph node of each treatment group were stimulated with PMA/ionomycin. The levels of cytokines were then compared between the groups. Treatment of GCSE significantly reduced the expression levels both in mRNA (Figure 4A) protein levels (Figure 4B) of pathogenic cytokines such as IL-4, IL-5, IL-10, IL-13 and IL-17 in a dose dependent manner. These results suggest that ameliorated AD symptoms by GCSE treatment is mediated by down-regulation of pathogenic cytokines. Interestingly, treatment of high dose of GCSE (10 mg) increased Foxp3 expression (Figure 4A). GCSE treatment also reduced the expression levels of IL-4 and IL-13 in B cells as compared with control mice. No difference was observed in the IL-5 expression levels between the groups. Moreover, reduction in IL-10 expression was observed in only in GCSE-10 mg treated group (Figure 4C).

### GCSE treatment increases Foxp3 expression in iTregs

**Figure 5:**
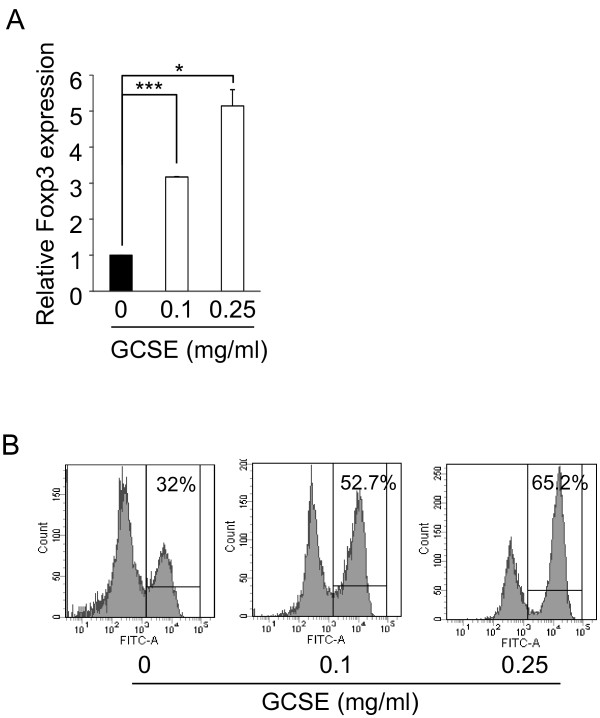
**Up-regulation of Foxp3 expression in iTreg by GCSE treatment.** CD4+ T cells isolated from the spleen and lymph node of 8 week old Foxp3-GFP knock in mice were stimulated in a medium supplemented with anti-CD3 (1 μg/ml) / CD28 Ab (3 μg/ml), anti-IL-4 Ab (10 μg/ml), anti-INF-γ Ab (10 μg/ml) and TGF-β (5 ng/ml) at day 1 and additional 50 U/ml rhIL-2 at day 3. Then iTreg cells were stimulated with various concentrations of GCSE in the presence of PMA (50 ng/ml)/ ionomycin (1 μM) for 12 hrs. **(A)** Relative expression levels of Foxp3 of GCSE treated samples were compared with control sample by qRT-PCR. Expression level of HPRT was used as an internal control. **(B)** GFP signal was measured as protein level of Foxp3 by flow cytometry. Error bars indicate SD. One (*), two (**), and three (***) indicate p < 0.05, p < 0.01, and p < 0.001 respectively. Data are representative of three independent experiments.

*In vivo* treatment of GCSE to AD-induced mice enhanced the Foxp3 expression in dLN CD4+ T cells (Figure 4A). In order to verify the effect of GCSE to Treg cells, we tested whether GCSE treatment could enhance the Foxp3, a marker of regulatory T cells, expression in *in vitro* differentiated inducible regulatory T cells (iTregs). CD4+ T cells isolated from Foxp3-GFP knock-in mice were cultured under iTreg differentiation condition [24] for 3 days, then, stimulated with various concentrations of GCSE in the presence of PMA/ionomycin for 12 hrs. As shown in Figure 5A, treatment of GCSE to iTreg cells significantly increased Foxp3 mRNA level in a dose-dependent manner (0.1 mg/ml; 3.2 fold, 0.25 mg/ml; 5.1 fold). Consistent with mRNA level result, Foxp3 protein level was also dose dependently up-regulated upon GCSE treatment (0 mg/ml; 32%, 0.1 mg/ml; 52.7%, 0.25 mg/ml; 65.2%) (Figure 5B). These results suggest that inhibitory effect of GCSE on the AD development could be mediated by induction of Foxp3 in regulatory T cells.

## Discussion

In this study, we identified a protective effect of GCSE against experimental AD progression and elucidated the underlying mechanism of action. Topical treatment of GCSE significantly mitigated the pathogenic symptoms of atopic dermatitis. GCSE treatment reduced serum IgE level and secreted IgE level in activated B cells. GCSE treatment also down-regulated the level of pathogenic cytokines by B cells and CD4+ T cells of AD mice.

Recently, we have demonstrated that Taglisodog-eum (TSE), a mixture of 11 herbs treatment, effectively suppressed the development of experimental AD by down-regulating pathogenic cytokines as well as IgE levels. Underlying mechanism of TSE was mainly mediated by reduction of NFκB (p65) transactivity in T cells and by reduction of *Aicda*-mediated IgE class switching in B cells [25]. Repeated treatment of TSE containing ointment effectively improved the symptoms of AD patients by reduction of SCORAD index as well as transepidermal water loss (TEWL) [26]. However TSE formula has a side effect such as irritation upon ointment treatment. Furthermore, it was very difficult to standardize the 11 complicated herbal extracts. To overcome those problems, we tested anti-atopic dermatitis effect of Gami-Cheongyeul-Sodok-Eum (GCSE), a modified formula of Cheongyeul-Sodok-Eum [16,17,27,28]. GCSE contains 9 kinds of oriental medicine extracts. Some components of GCSE have anti-inflammatory and anti-allergic effects [18-20,29,30]. Compared with each component of GCSE, GCSE showed the most potent inhibitory effect on IgE production (Additional file 1: Figure S1) as well as cytokine expression (Additional file 1: Figure S2 and Additional file 2).

Based on these results, we tested the immunomodulatory effect of GCSE on experimental atopic dermatitis. Several markers are employed to measure the severity of clinical symptoms of experimental atopic dermatitis including degree of scratching, pruritic skin lesion, and levels of pathogenic cytokines including IL-4, IL-5, IL-13 and IFN-γ. Serum IgE level is considered as one of the crucial markers of AD since about 70 ~ 80% of AD patients show significantly increased serum IgE level as compared with non-AD patients [23,31,32]. Prior to performing the *ex vivo* experiments with cells isolated from AD-induced mice, we firstly characterized CD4+ T cells and CD19+ B cells isolated from AD-induced mice by comparing with cells isolated from normal mice. As shown in Additional file 1: Figure S3, the expression levels of AD-related pathogenic cytokines such as IL-4, IL-5, IL-13, and IFN-γ in CD4+ T cells from AD-induced mice were significantly increased compared to that of normal CD4+ T cells. When we measured secreted IgE levels from CD19+ B cells, CD19+ B cells from AD-induced mice produced much higher level of IgE compared to that of normal mice. Next, we examined the effect of GCSE on CD4+ T cells and CD19+ B cells isolated from AD-induced mice. GCSE treatment significantly reduced IgE production by primary CD19+ B cells isolated from AD-induced mice (Figure 2B). GCSE treatment also suppressed the expression of AD-related pathogenic cytokines such as IL-4, IL-5, IL-13, IL-10, and IL-17 in CD4+ T cells isolated from AD-induced mice (Figure 2C-D). Topical application of GCSE significantly reduced AD symptoms and ear thickness (Figure 3A-B) and it significantly decreased tissue infiltration of lymphocytes (Figure 3C). On the aspect of B cells as an IgE producer, it is quite notable that GCSE treatment significantly reduced serum IgE levels (Figure 3D) as well as secretion of IgE in the B cell culture supernatant in a dose-dependent manner (Figure 3E). Atopic dermatitis has been thought as a typical Th2 type immune disorder that expresses high levels of Th2 type cytokines such as IL-4, IL-5, and IL-13. However, recently, several groups suggested that pro-inflammatory Th1 or Th17 type immune responses also play key roles in the maintenance of chronic stage of AD [6,8,33]. IL-4, IL-5 and IL-13 are typical Th2 type cytokines that stimulate Th2 differentiation and IgE production by B cells [10,34]. IFN-γ is a typical Th1 type cytokines that upregulates the expression of CCL17 (TARC) and CCL22 (MDC), which recruit Th2 type cytokines to the inflamed site [35,36]. IL-17 coordinates local tissue inflammation through upregulation of pro-inflammatory cytokines (IL-6, TNF-α), neutrophil-mobilizing cytokines (GM-CSF, MIP-2/CXCL2), chemokines (MCP-1/CCL2, MIP-3α/CCL20) [37,38]. These effector molecules collectively enable migration of activated T cells through extracellular matrix. Therefore, down-regulation of Th1/Th17 and Th2 type of immune responses is necessary to successfully modulate atopic dramatis. In this aspect, it is quite notable that GCSE treatment significantly down-regulated both Th2 cytokines (IL-4, IL-5, IL-10, and IL-13) and Th1/Th17 type pro-inflammatory cytokines such as IFN-γ and IL-17 (Figure 4A-B). High levels of IL-4 and IL-13 produced by CD4+ T cells induce a class switching of plasma cells to produce IgE [39,40]. IL-13 produced by B cells also plays a crucial role in IgE production in an autocrine manner [10]. Down-regulation of IL-4 and IL-13 by CD19+ B cells (Figure 4C) and CD4+ T cells (Figure 4A) upon GCSE treatment may cause the inhibition of the IgE production. Furthermore, high concentration GCSE treatment (GCSE-10 mg) increased Foxp3 expression. The forkhead family protein Foxp3 is a transcription factor that is highly expressed in CD4+ regulatory T cells (Tregs). Foxp3 is a key regulator of T cell tolerance and plays a pivotal role to the development and function of Tregs [41]. Interestingly, addition of GCSE to iTreg cells significantly increased mRNA as well as protein level of Foxp3 in a dose-dependent manner (Figure 5). This result indicates that GCSE may have a potential to generate iTregs. However, further studies are required to identify the exact component of GCSE that has iTreg inducing activity.

## Conclusions

In conclusion, we have demonstrated that topical treatment of GCSE ameliorated the progression of experimental atopic dermatitis by reducing serum IgE and AD-associated pathogenic cytokines levels while increasing Foxp3 level. Our study collectively suggests the beneficial effect of GCSE treatment in inhibiting the progression of atopic dermatitis.

## Abbreviations

GCSE: Gami-Cheongyeul-Sodok-Eum; Cont: Control; LPS: Lipopolysachharide; PMA: Phorbol 12-myristate 13-acetate; AD: Atopic dermatitis; Th1 & Th2: T helper 1 and T helper 2; IL-4: Interleukin 4; IL-5: Interleukin 5; IL-10: Interleukin 10; IL-13: Interleukin 13; IL-17: Interleukin 17; IFN-γ: Interferon gamma; TNF-α: Tumor necrosis factor alpha; Foxp3: Forkhead box P3; TGF-β: Transforming growth factor beta; DNCB: 2, 4-dinitrocholorobenzene; iTreg: inducible regulatory T cell.

## Competing interests

The authors declare that they have no competing interests.

## Authors’ contributions

S-HI and Y-BY designed the research; J-SH, J-EK and Y-BY conducted research; J-SH and S-HI analyzed data; J-SH and S-HI wrote the paper; Y-BY and S-HI had primary responsibility for final content. All authors read and approved the final manuscript.

## Pre-publication history

The pre-publication history for this paper can be accessed here:

http://www.biomedcentral.com/1472-6882/13/312/prepub

## Supplementary Material

Additional file 1: Figure S1 Effect of GCSE and its components on IgE production. **Figure S2.** Inhibitory effect of GCSE on cytokine production. **Figure S3.** Characteristics of CD4+ T cells and CD19+ B cells from normal and AD induced mice. Click here for file

Additional file 2 Topical application of Gami-Cheongyeul-Sodok-Eum (GCSE). Click here for file
