# Supplementary material for: Modulation of experimental atopic dermatitis by topical application of Gami-Cheongyeul-Sodok-Eum
Source: BMC Complement Altern Med. 2013 Nov 11;13:312. doi: 10.1186/1472-6882-13-312 (PMC3832229; doi:10.1186/1472-6882-13-312)
Supplement: Additional file 1: Figure S1 — Effect of GCSE and its components on IgE production. Figure S2. Inhibitory effect of GCSE on cytokine production. Figure S3. Characteristics of CD4+ T cells and CD19+ B cells from normal and AD induced mice. [file 1472-6882-13-312-S1.pptx]

## Slide 1
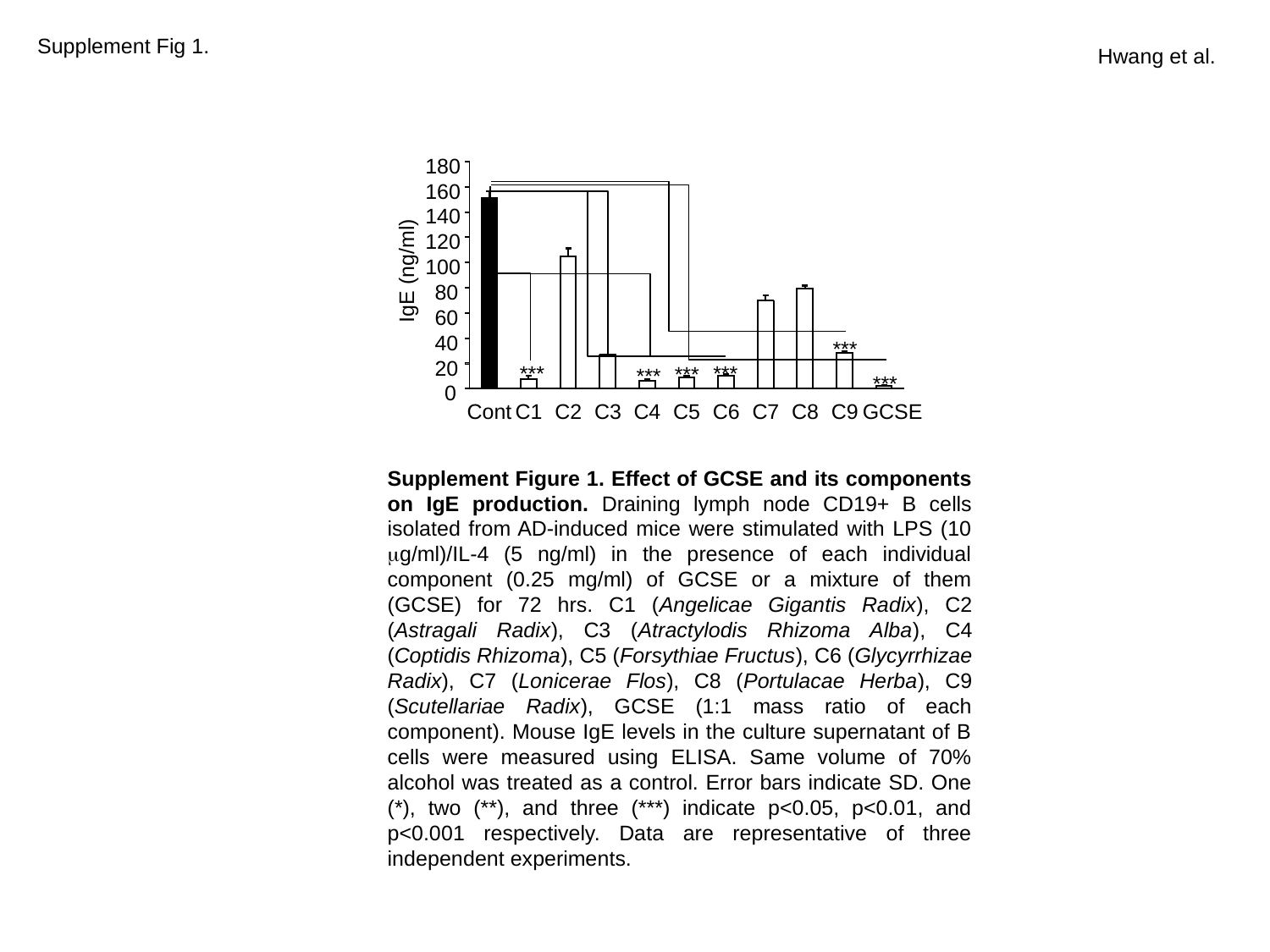

Supplement Fig 1.
Hwang et al.
180
160
140
120
100
IgE (ng/ml)
80
60
40
20
0
Cont
C1
C2
C3
C4
C5
C6
C7
C8
C9
GCSE
***
***
***
***
***
***
Supplement Figure 1. Effect of GCSE and its components on IgE production. Draining lymph node CD19+ B cells isolated from AD-induced mice were stimulated with LPS (10 mg/ml)/IL-4 (5 ng/ml) in the presence of each individual component (0.25 mg/ml) of GCSE or a mixture of them (GCSE) for 72 hrs. C1 (Angelicae Gigantis Radix), C2 (Astragali Radix), C3 (Atractylodis Rhizoma Alba), C4 (Coptidis Rhizoma), C5 (Forsythiae Fructus), C6 (Glycyrrhizae Radix), C7 (Lonicerae Flos), C8 (Portulacae Herba), C9 (Scutellariae Radix), GCSE (1:1 mass ratio of each component). Mouse IgE levels in the culture supernatant of B cells were measured using ELISA. Same volume of 70% alcohol was treated as a control. Error bars indicate SD. One (*), two (**), and three (***) indicate p<0.05, p<0.01, and p<0.001 respectively. Data are representative of three independent experiments.

## Slide 2
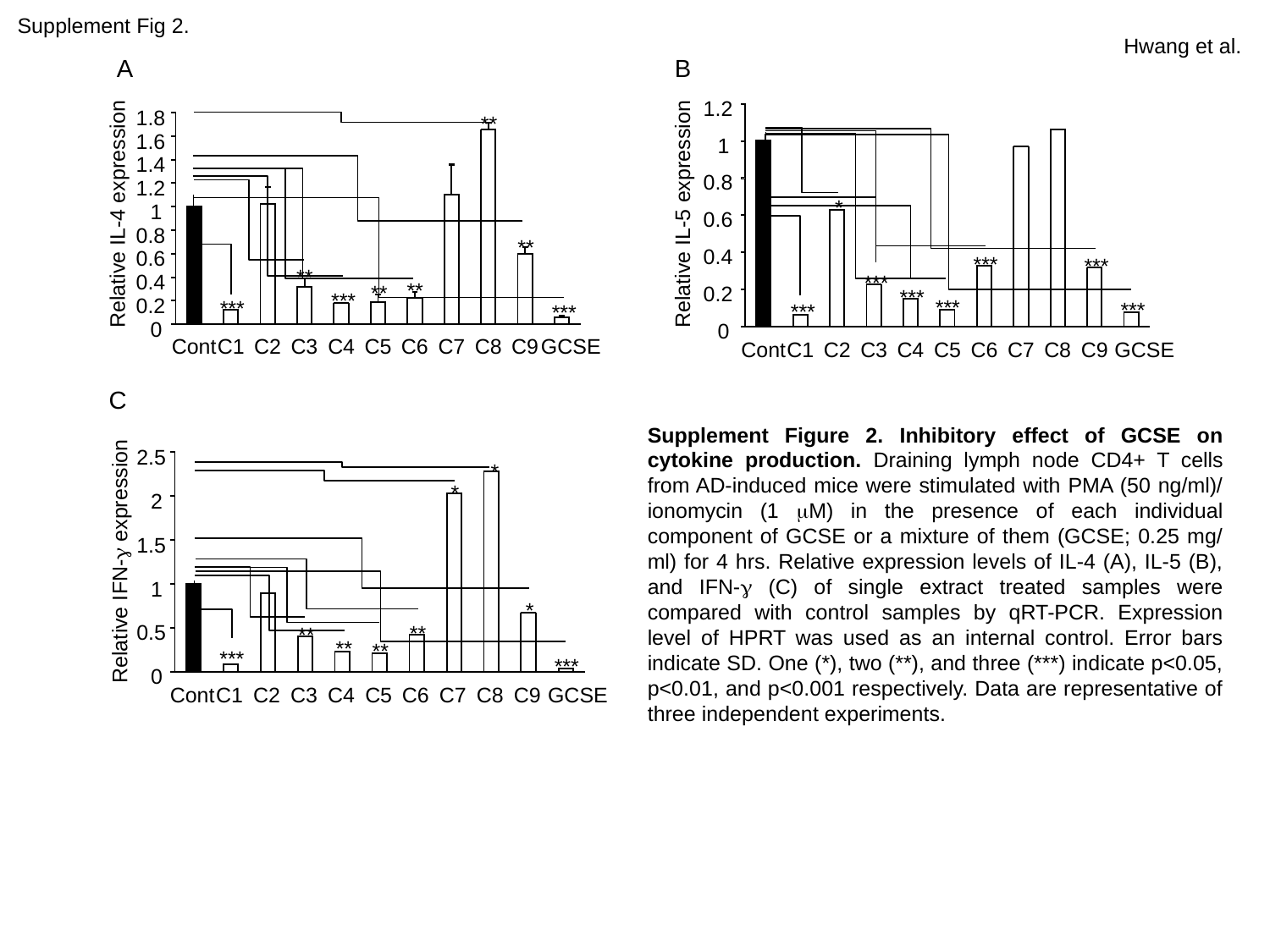

Supplement Fig 2.
Hwang et al.
B
A
1.2
1
0.8
Relative IL-5 expression
0.6
0.4
0.2
0
Cont
C1
C2
C3
C4
C5
C6
C7
C8
C9
GCSE
*
***
***
***
***
***
***
***
1.8
1.6
1.4
1.2
1
Relative IL-4 expression
0.8
0.6
0.4
0.2
0
C1
C2
C3
C4
C5
C6
C7
C8
C9
GCSE
Cont
**
**
**
**
**
***
***
***
2.5
2
1.5
Relative IFN-g expression
1
0.5
0
C1
C2
C3
C4
C5
C6
C7
C8
C9
GCSE
Cont
*
*
*
**
**
**
**
***
***
C
Supplement Figure 2. Inhibitory effect of GCSE on cytokine production. Draining lymph node CD4+ T cells from AD-induced mice were stimulated with PMA (50 ng/ml)/ionomycin (1 mM) in the presence of each individual component of GCSE or a mixture of them (GCSE; 0.25 mg/ml) for 4 hrs. Relative expression levels of IL-4 (A), IL-5 (B), and IFN-g (C) of single extract treated samples were compared with control samples by qRT-PCR. Expression level of HPRT was used as an internal control. Error bars indicate SD. One (*), two (**), and three (***) indicate p<0.05, p<0.01, and p<0.001 respectively. Data are representative of three independent experiments.

## Slide 3
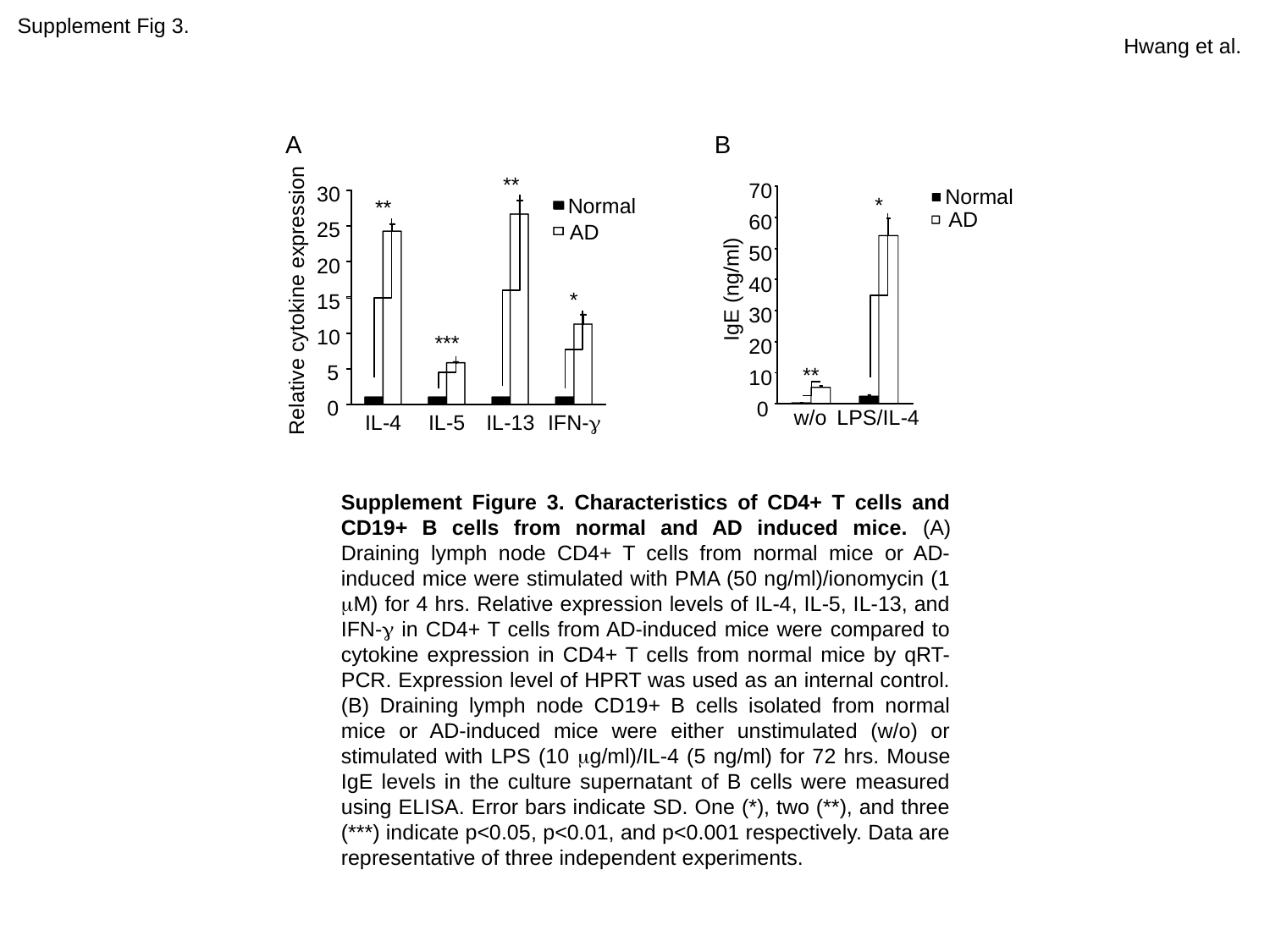

Supplement Fig 3.
Hwang et al.
A
**
30
Normal
25
AD
20
Relative cytokine expression
15
10
5
0
IL-4
IL-5
IL-13
IFN-g
**
*
***
B
70
Normal
AD
60
50
IgE (ng/ml)
40
30
20
10
0
w/o
LPS/IL-4
*
**
Supplement Figure 3. Characteristics of CD4+ T cells and CD19+ B cells from normal and AD induced mice. (A) Draining lymph node CD4+ T cells from normal mice or AD-induced mice were stimulated with PMA (50 ng/ml)/ionomycin (1 mM) for 4 hrs. Relative expression levels of IL-4, IL-5, IL-13, and IFN-g in CD4+ T cells from AD-induced mice were compared to cytokine expression in CD4+ T cells from normal mice by qRT-PCR. Expression level of HPRT was used as an internal control. (B) Draining lymph node CD19+ B cells isolated from normal mice or AD-induced mice were either unstimulated (w/o) or stimulated with LPS (10 mg/ml)/IL-4 (5 ng/ml) for 72 hrs. Mouse IgE levels in the culture supernatant of B cells were measured using ELISA. Error bars indicate SD. One (*), two (**), and three (***) indicate p<0.05, p<0.01, and p<0.001 respectively. Data are representative of three independent experiments.
