# Supplementary material for: Modulation of experimental atopic dermatitis by topical application of Gami-Cheongyeul-Sodok-Eum
Source: BMC Complement Altern Med. 2013 Nov 11;13:312. doi: 10.1186/1472-6882-13-312 (PMC3832229; doi:10.1186/1472-6882-13-312)
Supplement: Additional file 2 — Topical application of Gami-Cheongyeul-Sodok-Eum (GCSE). [file 1472-6882-13-312-S2.pdf]

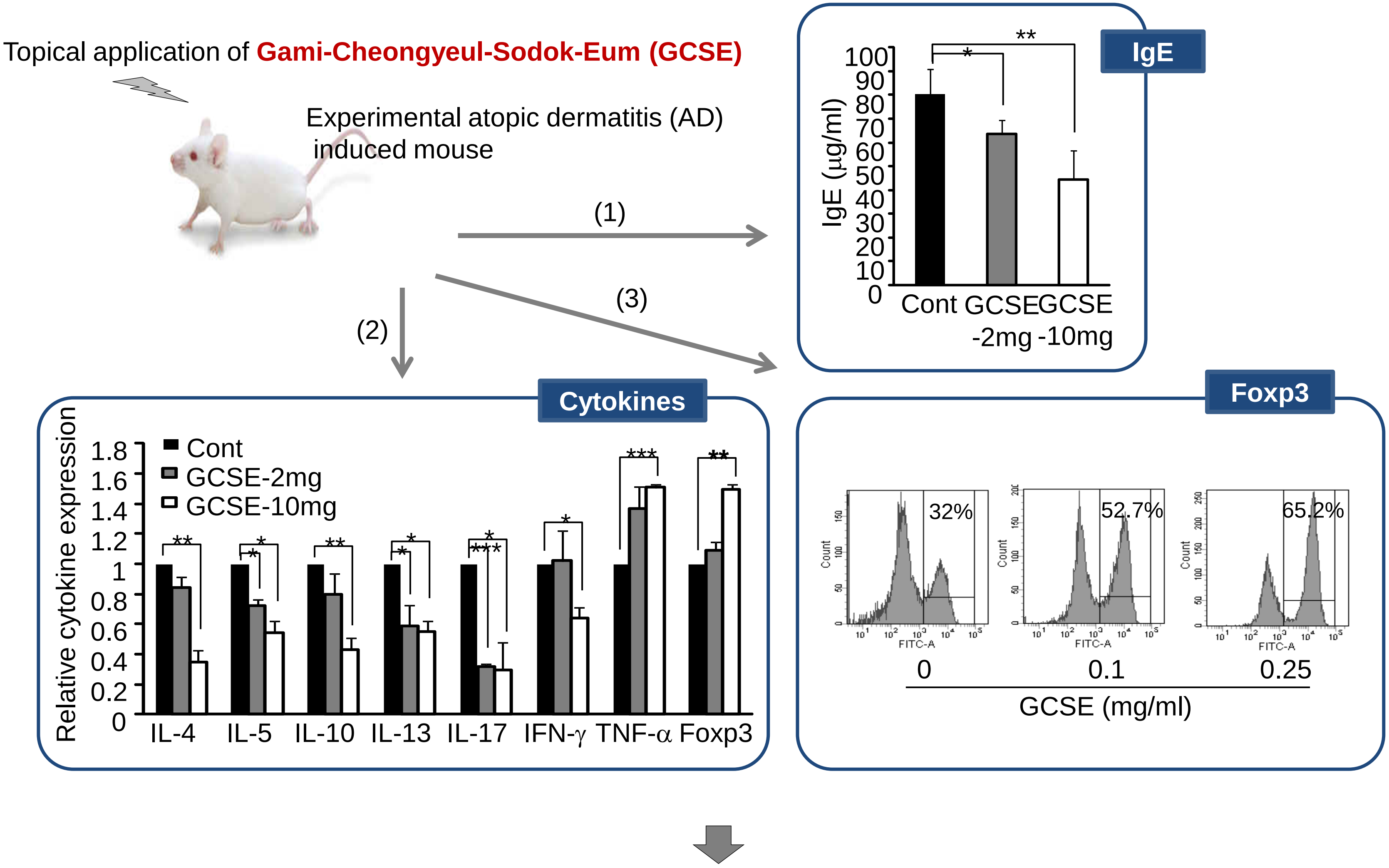

Progression of AD development was inhibited by topical application of GCSE via (1) reduction of serum total IgE, (2) down regulation of AD-pathogenic cytokines and (3) up-regulation of Foxp3.
